# Impaired mitochondrial oxidative phosphorylation capacity in epicardial adipose tissue is associated with decreased concentration of adiponectin and severity of coronary atherosclerosis

**DOI:** 10.1038/s41598-019-40419-7

**Published:** 2019-03-05

**Authors:** Takayuki Nakajima, Takashi Yokota, Yasushige Shingu, Akira Yamada, Yutaka Iba, Kosuke Ujihira, Satoru Wakasa, Tomonori Ooka, Shingo Takada, Ryosuke Shirakawa, Takashi Katayama, Takaaki Furihata, Arata Fukushima, Ryosuke Matsuoka, Hiroshi Nishihara, Flemming Dela, Katsuhiko Nakanishi, Yoshiro Matsui, Shintaro Kinugawa

**Affiliations:** 1 Department of Cardiovascular Medicine, Faculty of Medicine and Graduate School of Medicine, Hokkaido University, Sapporo, Japan; 2 Department of Cardiovascular and Thoracic Surgery, Faculty of Medicine and Graduate School of Medicine, Hokkaido University, Sapporo, Japan; 3 Department of Cardiovascular Surgery, Teine Keijinkai Hospital, Sapporo, Japan; 4 Division of Clinical Cancer Genomics, Hokkaido University Hospital, Sapporo, Japan; 5 Division of Clinical Cancer Genomics, Hokkaido Cancer Center, Sapporo, Japan; 6 Xlab, Center for Healthy Aging, Department of Biomedical Sciences, University of Copenhagen, Copenhagen, Denmark; 7 Department of Geriatrics, Bispebjerg-Frederiksberg University Hospital, Copenhagen, Denmark

## Abstract

Epicardial adipose tissue (EAT), a source of adipokines, is metabolically active, but the role of EAT mitochondria in coronary artery disease (CAD) has not been established. We investigated the association between EAT mitochondrial respiratory capacity, adiponectin concentration in the EAT, and coronary atherosclerosis. EAT samples were obtained from 25 patients who underwent elective cardiac surgery. Based on the coronary angiographycal findings, the patients were divided into two groups; coronary artery disease (CAD; n = 14) and non-CAD (n = 11) groups. The mitochondrial respiratory capacities including oxidative phosphorylation (OXPHOS) capacity with non-fatty acid (complex I and complex I + II-linked) substrates and fatty acids in the EAT were significantly lowered in CAD patients. The EAT mitochondrial OXPHOS capacities had a close and inverse correlation with the severity of coronary artery stenosis evaluated by the Gensini score. Intriguingly, the protein level of adiponectin, an anti-atherogenic adipokine, in the EAT was significantly reduced in CAD patients, and it was positively correlated with the mitochondrial OXPHOS capacities in the EAT and inversely correlated with the Gensini score. Our study showed that impaired mitochondrial OXPHOS capacity in the EAT was closely linked to decreased concentration of adiponectin in the EAT and severity of coronary atherosclerosis.

## Introduction

Growing evidence suggests that the accumulation of ectopic fat such as visceral abdominal fat and intramyocellular lipid is closely linked to insulin resistance and atherosclerosis^1^. Cardiac adipose tissue is also recognized as an ectopic fat that is divided into two parts of adipose tissue by the pericardium; epicardial adipose tissue (EAT) and pericardial adipose tissue (PAT). The EAT surrounds the heart and the coronary arteries within the pericardium, whereas the PAT surrounds the heart but is distributed outside the pericardium. The EAT constitutes approximately 20% of the total ventricular weight of a healthy adult^2^. Because of its anatomical proximity to the coronary artery, the role of EAT in coronary artery disease (CAD) has drawn much attention^3,4^.

Although several investigations demonstrated that EAT volume is increased in patients with cardiovascular disease in association with disease severity^5–7^, some clinical studies did not find significant associations between EAT volume and severity of coronary artery stenosis^8,9^. Accordingly, a question about whether increased EAT volume is linked to coronary artery stenosis ‘directly’ or in combination with other risk factors such as functional changes of the EAT arises.

The EAT is metabolically active; it secrets various bioactive molecules (including adipokines) that are related to energy metabolism and inflammation^10^. Because there is no fibrous fascial layer that impedes the diffusion of these adipokines from the EAT to the coronary arteries, functional changes in the EAT may directly affect coronary artery sclerosis via a paracrine pathway^4^. Mitochondria play a key role in the maintenance of cellular function as a main energy source, and mitochondrial dysfunction including decreased mitochondrial respiration is thought to be involved in the pathogenesis of cardiovascular disease^11–13^. An *in vitro* study has shown that the lowered mitochondrial respiratory capacity results in decreased secretion of adiponectin, an anti-inflammatory and anti-atherogenic adipokine, from adipocytes^14^, which raises the possibility that lowered EAT mitochondrial respiratory capacity may result in reduced secretion of adiponectin from the EAT, and the lack of adiponectin in the EAT may contribute to the development of coronary atherosclerosis.

However, there is no study that investigated EAT mitochondrial respiratory capacity in human. Here we examined: (1) whether EAT mitochondrial respiratory capacity was lowered in CAD patients, (2) whether lowered EAT mitochondrial respiratory capacity was associated with protein levels of adiponectin in the EAT and severity of coronary artery stenosis, and (3) whether protein levels of adiponectin in the EAT was associated with severity of coronary artery stenosis.

## Results

### Patient characteristics

The characteristics of the patients in the CAD and non-CAD groups are summarized in Table 1. There was no significant difference in age, gender, body mass index, and visceral abdominal fat area between the CAD and non-CAD groups. The left ventricular ejection fraction (LVEF) evaluated by echocardiography before cardiac surgery was comparable between the groups. CAD patients had significantly higher prevalences of diabetes and dyslipidemia compared to non-CAD patients. The majority of CAD patients (86%) had multivessel CAD.

**Table 1:** Patient characteristics.

	Non-CAD (n = 11)	CAD (n = 14)	*P* value
Age, yrs	68 ± 14	68 ± 11	0.99
Male/female	4/7	8/6	0.30
BMI, kg/m^2^	23.6 ± 4.2	24.3 ± 3.6	0.66
Visceral abdominal fat area, cm^2^	62.1 ± 49.9	96.9 ± 44.0	0.08
LVEF, %	56 ± 16	50 ± 15	0.37
CAD			
1-vessel disease	0 (0)	2 (14)	
2-vessel disease	0 (0)	4 (29)	
3-vessel disease	0 (0)	8 (57)	
Complications			
Hypertension	6 (55)	10 (71)	0.38
Diabetes mellitus	2 (18)	11 (79)	0.003
Dyslipidemia	2 (18)	13 (93)	<0.001
Medication			
β-blocker	4 (36)	8 (57)	0.30
ACE inhibitor or ARB	4 (36)	9 (64)	0.24
Statins	2 (18)	14 (100)	<0.001
Antidiabetics	2 (18)	6 (43)	0.19
HbA_1c_, %	5.6 ± 0.3	7.2 ± 1.3	<0.001
Triglyceride, mmol/L	1.58 ± 1.31	1.32 ± 0.12	0.63
HDL-cholesterol, mmol/L	1.47 ± 0.50	1.26 ± 0.29	0.22
LDL-cholesterol, mmol/L	2.58 ± 0.95	2.46 ± 0.79	0.75
Serum adiponectin, µg/mL	12.3 ± 5.6	4.6 ± 4.0	<0.001

Values are mean ± SD or n (%). ACE, angiotensin converting enzyme; ARB, angiotensin receptor blocker; BMI, body mass index; CAD, coronary artery disease; HbA1c, hemoglobin A1c; HDL, high-density lipoprotein; LDL, low-density lipoprotein; LVEF, left ventricular ejection fraction.

Hemoglobin A1c values were significantly higher and serum levels of adiponectin were significantly lower in CAD patients. In contrast, there was no significant difference in fasting triglyceride or low- or high-density lipoprotein cholesterol levels between the groups, although more CAD patients than non-CAD patients were taking statins.

### Mitochondrial respiratory capacity in the EAT

**Figure 1:**
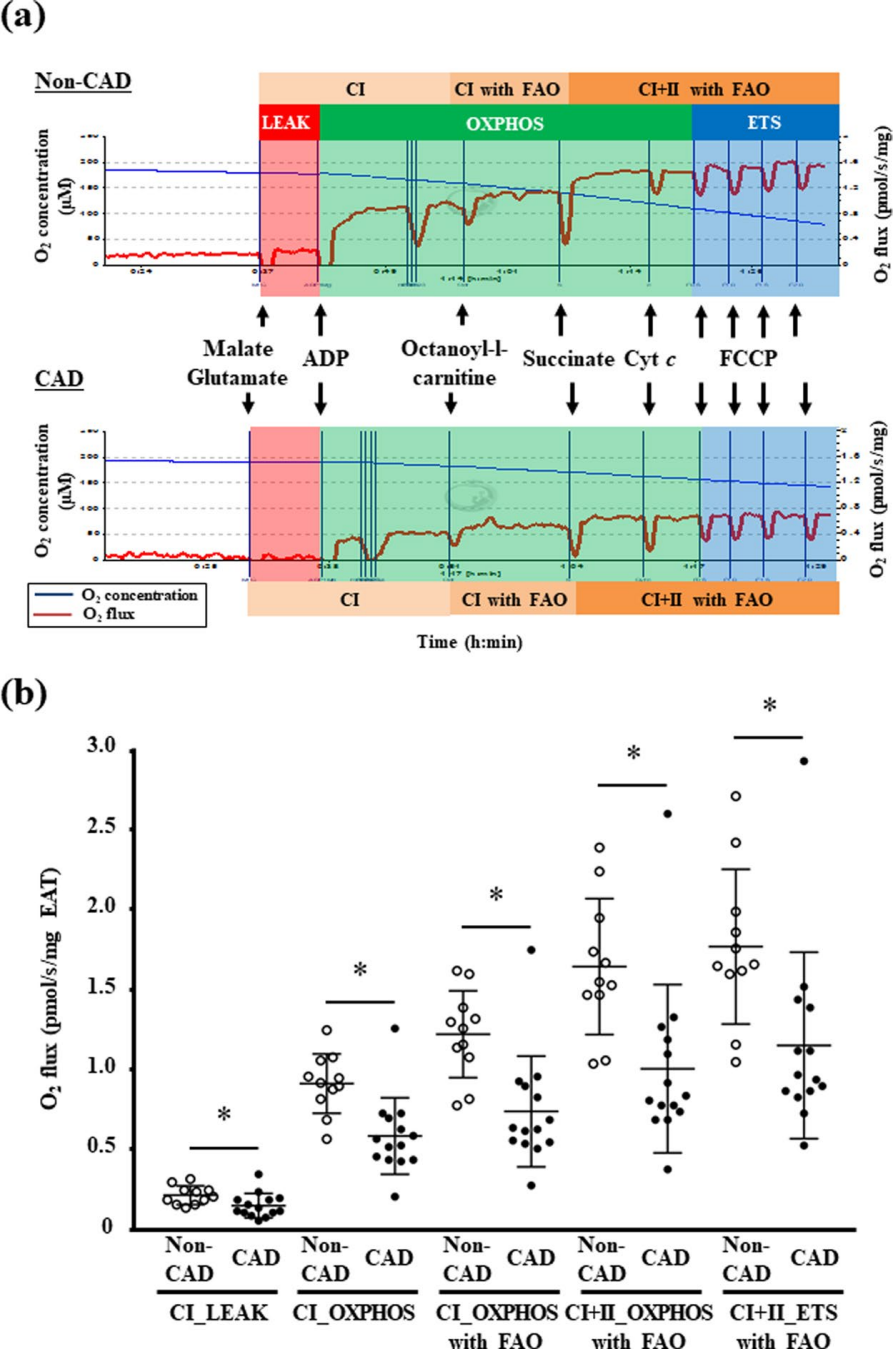
The mitochondrial respiratory capacity in the EAT. (**a**) Representative graphs of mitochondrial respiratory capacity in the EAT in the non-coronary artery disease (non-CAD, n = 11) and CAD patients (n = 14). (**b**) The mitochondrial respiratory capacity at each state with non-fatty acid and fatty acid substrates in the EAT was lowered in the CAD group. Bar: mean ± SD. *P < 0.05. CI, complex I-linked substrates; CI + II, complex I + II-linked substrates; ETS, maximal electron transfer system capacity; FAO, fatty acid oxidation; LEAK, leak-state respiration (non-ADP stimulated respiration); OXPHOS, oxidative phosphorylation capacity (ADP-stimulated respiration).

Representative graphs of the mitochondrial respiratory capacity in the permeabilized EAT in the CAD and non-CAD groups are provided in Fig. 1a. CAD patients showed a lower LEAK respiration (i.e., non-ADP stimulated respiration) with complex I-linked substrates in their EAT compared to non-CAD patients (Fig. 1b). The mitochondrial OXPHOS capacity (i.e., ADP-stimulated respiration) with complex I-linked substrates in the EAT was significantly reduced in the CAD group compared to the non-CAD group (Fig. 1b). CAD patients had lower capacities of complex I-linked and complex I + II-linked OXPHOS under the existence of fatty acids in the EAT compared to non-CAD patients (Fig. 1b). Moreover, the maximal electron transfer system (ETS) capacity in the EAT evaluated after FCCP titration was significantly decreased in the CAD group compared to the non-CAD group (Fig. 1b).

### Association between the mitochondrial respiratory capacity in the EAT and the severity of coronary artery stenosis

**Figure 2:**
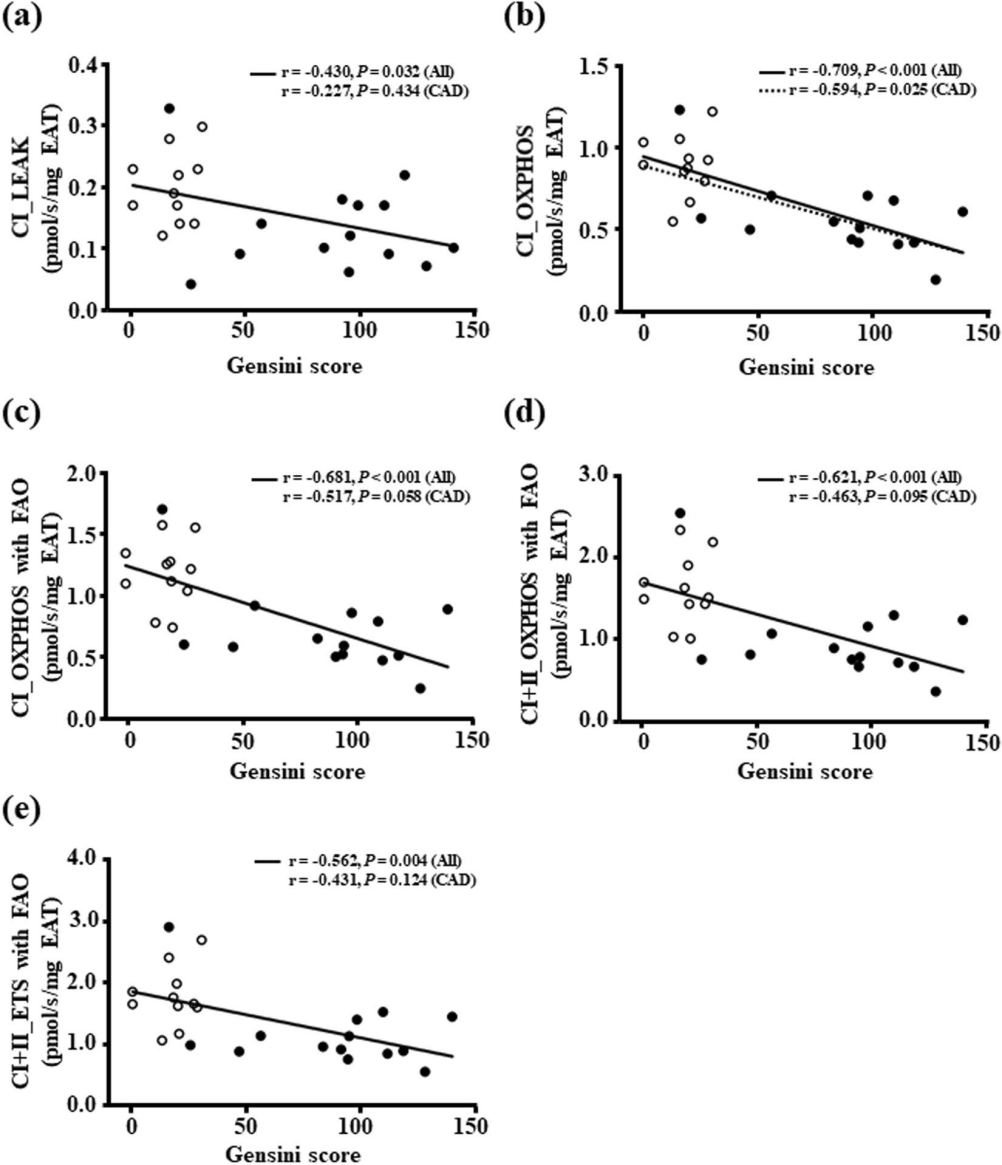
The association between the mitochondrial respiratory capacity in the EAT and the severity of coronary artery sclerosis. White and black circles indicate non-CAD (n = 11) and CAD patients (n = 14), respectively. A solid line indicates a significant correlation in all patients and a dashed line indicates a significant correlation only in CAD patients. Abbreviations are explained in the Fig. 1 legend.

We examined whether the lowered mitochondrial respiratory capacity in the EAT was associated with the Gensini score, a parameter of the severity of coronary artery stenosis. Our analyses of all patients demonstrated that the mitochondrial respiratory capacities at all states with non-fatty acid and fatty acid substrates in the EAT were inversely correlated with the Gensini score (Fig. 2a–e). In addition, when the analysis was performed only in CAD patients, the Gensini score had a significant and inverse correlation with only EAT mitochondrial OXPHOS capacity with complex I-linked substrates (Fig. 2b).

### Protein content of the adiponectin in the EAT

**Figure 3:**
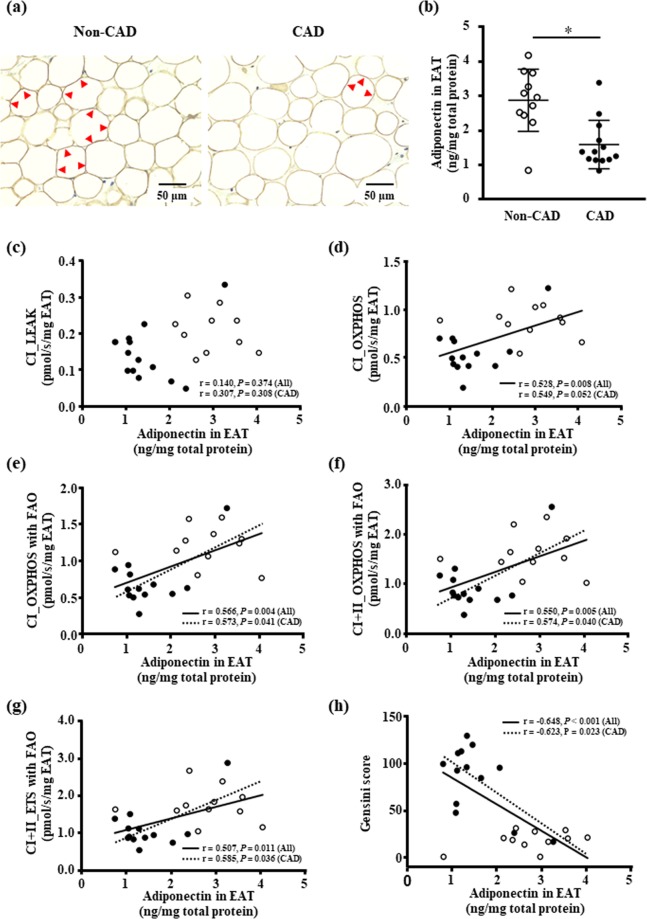
The protein content of adiponectin in the EAT. (**a**) Representative images of immunohistochemical staining of adiponectin in the EAT of the non-CAD and CAD patients. Adiponectin is stained in dark brown in the cytosol around the lipid droplet in the adipocyte of the EAT (see red arrows), and intensity of adiponectin staining appears to be weak in a CAD patient compared to a non-CAD patient. **(b)** The protein levels of adiponectin in EAT in non-CAD (n = 11) and CAD patients (n = 13). Bar: mean ± SD. *P < 0.05. **(c**–**g)** The association the protein content of adiponectin and the mitochondrial respiratory capacity in the EAT. (**h**) The association between the protein content of adiponectin and the severity of coronary artery stenosis. White and black circles indicate non-CAD (n = 11) and CAD patients (n = 13), respectively. A solid line indicates a significant correlation in all patients and a dashed line indicates a significant correlation only in CAD patients. Abbreviations are explained in the Fig. 1 legend.

The results of our analysis of the adiponectin synthesis in the EAT are illustrated in Fig. 3a,b. The representative immunohistochemical staining shows decreased adiponectin staining around the lipid droplet in adipocytes of the EAT in the CAD compared to non-CAD groups (Fig. 3a). The adiponectin protein level of the EAT was significantly decreased in CAD patients compared to non-CAD patients (Fig. 3b).

### Association between protein content of adiponectin in the EAT and the mitochondrial respiratory capacity in the EAT or the severity of coronary artery stenosis

Both the mitochondrial OXPHOS capacity and the maximal ETS capacity with non-fatty acid and fatty acid substrates were positively correlated with the protein levels of adiponectin in the EAT (Fig. 3d–g). In contrast, there was no correlation between the mitochondrial respiration in the LEAK with complex I-linked substrates and the adiponectin concentration in the EAT (Fig. 3c). In addition, the protein levels of adiponectin in the EAT had an inverse correlation with the Gensini score in all patients (Fig. 3h). The protein levels of adiponectin in the EAT were positively correlated with the mitochondrial OXPHOS capacity with complex I and fatty acid-linked substrates (Fig. 3e) and with complex I + II and fatty acid-linked substrates (Fig. 3f), or the maximal ETS capacity with non-fatty acid and fatty acid substrates (Fig. 3g) even in CAD patients. There was also a significant correlation between the Gensini score and the pretein content of adiponectin in the EAT in CAD patients (Fig. 3h).

### EAT volume

**Figure 4:**
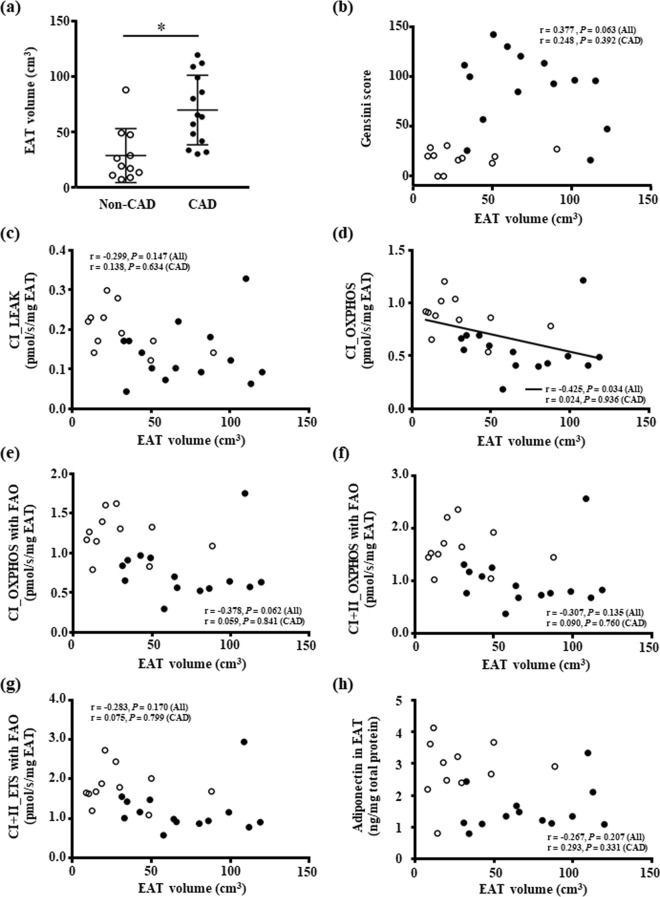
The EAT volume. (**a**) Increased EAT volume in the CAD patients. Bar: mean ± SD. *P < 0.05. (**b**) The association between the EAT volume and the severity of coronary artery stenosis. (**c**–**g**) The association between the EAT volume and the mitochondrial respiratory capacity in the EAT. (**h**) The association between the EAT volume and the protein level of adiponectin in the EAT. White and black circles indicate non-CAD (n = 11) and CAD patients (n = 14 except for (**h**) [n = 13]), respectively. A solid line indicates a significant correlation in all patients. Abbreviations are explained in the Fig. 1 legend.

The EAT volume of CAD patients was greater than that of non-CAD patients (Fig. 4a), but the EAT volume was not correlated with the Gensini score (Fig. 4b). The EAT volume was inversely correlated with the mitochondrial complex I-linked OXPHOS capacity in the EAT in all patients (Fig. 4d), but there was no significant correlation between the EAT volume and other mitochondrial respiratory capacities in the EAT (Fig. 4c,e–g). In addition, there was no significant correlation between the EAT volume and adiponectin levels in the EAT (Fig. 4h). The EAT volume was not correlated with any parameters when the analysis was performed only in CAD patients.

## Discussion

The main finding of the present study is that the mitochondrial respiratory capacity in the EAT was significantly lowered in CAD patients compared to non-CAD patients who underwent cardiac surgery. The lowered EAT mitochondrial respiratory capacity was closely associated with the severity of coronary artery stenosis evaluated by the Gensini score calculated based on the findings of coronary angiography. In contrast, there was no significant correlation between the EAT volume and the severity of coronary artery stenosis, although the EAT volume was larger in CAD patients compared to non-CAD patients. Intriguingly, protein content of adiponectin in the EAT was decreased in CAD patients, and this was associated with the lowered mitochondrial respiratory capacity and the severity of coronary artery stenosis in this tissue.

We observed that CAD patients had lower mitochondrial respiratory capacity with non-fatty acid and fatty acid substrates in their EAT. To our knowledge, this is the first study to demonstrate the mitochondrial respiratory capacity in human EAT. Unlike metabolically active organs such as the heart and skeletal muscle, the bioenergetics in adipose tissue have been poorly studied in part because of the smaller mitochondrial content in such tissue. However, recent advances in high-resolution respirometry and progress in the methodology of the measurement of mitochondrial respiratory capacity have enabled us to accurately evaluate mitochondrial function with a small sample size even in metabolically less-active tissues like the adipose tissue than the heart and skeletal muscle which have continuously high energy requirements for muscle contraction.

Kraunsøe *et al*. demonstrated that the visceral abdominal fat had a higher level of mitochondrial activity than the subcutaneous fat in same subjects^15^, which seems reasonable because the visceral abdominal fat is an important source of a number of bioactive molecules including adipokines. In humans, the EAT is also an active endocrine organ that secrets a greater amount of adipokines compared to the subcutaneous fat^16^. Thus, the mitochondrial respiratory capacity in the adipose tissues including the EAT may contribute to the metabolic activity of these tissues and may play a role in the maintenance of adipose tissue function such as the secretion of adipokines and energy homeostasis (e.g., storage of fatty acids).

In our study, CAD patients had a higher prevalence of type 2 diabetes than non-CAD patients. A previous study has shown that gene expression related to the mitochondrial ETS including complex I and II is downregulated in the visceral abdominal fat in individuals with type 2 diabetes, which might be linked to insulin resistance via metabolic disturbance in this fat tissue^17^. Accordingly, type 2 diabetes might be related to the lowered mitochondrial respiratory capacity in the EAT in CAD patients.

In the adult heart, EAT commonly surrounds coronary arteries, and there is no structure such as fibrous fascia that separates a coronary artery and its surrounding adipose tissue^4^. Our present findings demonstrated that the EAT volume in CAD patients was greater than that of non-CAD patients, independently of the amount of visceral abdominal fat, which is consistent with previous studies^7^. However, we and others showed that there was no correlation between the EAT volume and the severity of coronary artery stenosis^9^. Accordingly, changes in the quality of EAT rather than changes in the quantity of EAT may be a stronger risk factor for coronary artery sclerosis. Indeed, we found that the impaired mitochondrial respiratory capacity in the EAT is closely correlated with the severity of coronary artery stenosis. Yudkin *et al*. proposed that there is a contribution of the local fat depot to the pathogenesis of atherosclerosis in neighboring arteries via a paracrine pathway^18^, which supports the hypothesis that impaired EAT mitochondrial respiration directly or indirectly contributes to coronary atherosclerosis.

We measured the protein content of adiponectin in the EAT samples, and it was significantly decreased in CAD patients, which is consistent with previous studies^19,20^. Importantly, the protein levels of the adiponectin in the EAT samples were positively correlated with the mitochondrial OXPHOS capacity with non-fatty acid and fatty acid substrates in the EAT. An *in vitro* study demonstrated that the lowered mitochondrial respiration in adipocytes resulted in reduced adiponectin secretion from these cells^14^, suggesting that mitochondrial OXPHOS capacity in the EAT might regulate adiponectin secretion from the EAT. We showed that adiponectin concentration in the EAT had an inverse correlation with severity of coronary atherosclerosis. Because adiponectin has an anti-atherogenic and anti-inflammatory effect on the blood vessels, the reduced protein content of adiponectin in the EAT may play a role in the progression of coronary atherosclerosis.

Other possible mechanisms can be suggested to explain the role of the functional change of EAT in the pathogenesis and progression of CAD. It was reported that the gene expression of proinflammatory cytokines such as tumor necrosis factor-alpha (TNF-α), interleukin (IL)-6, and monocyte chemoattractant protein-1 (MCP-1) in the EAT is increased in CAD patients^16^. Increased proinflammatory cytokine release from the EAT may lead to the aggravation of inflammation in neighboring coronary arteries and thus subsequently stimulate atherosclerosis^16,18^. A phenotype change of M1 and M2 macrophages infiltrating into the EAT may also contribute to coronary atherosclerosis via the cytokine release from the EAT^21^.

We have some study limitations that should be acknowledged. First, our sample size was small. Additional studies with larger sample sizes are needed to determine whether impaired mitochondrial respiratory capacity in the EAT can be an independent cardiovascular risk factor. Second, some of the correlations between the mitochondrial respiratory capacity in the EAT and the severity of coronary artery stenosis were not significant when the analysis was performed only in CAD patients. Because most of our present CAD patients had multivessel disease and thus the range of Gensini scores in the CAD group was small, we could not detect a significant correlation. Finally, we cannot conclude that there is a causal relationship between the severity of coronary artery stenosis or protein levels of adiponectin and the mitochondrial respiratory capacity in the EAT.

In summary, the mitochondrial respiratory capacity in the EAT was significantly lowered in CAD patients. The lowered mitochondrial respiratory capacity in the EAT, but not the increased EAT volume, was closely correlated with the severity of coronary artery stenosis. In addition, the protein level of the adiponectin in the EAT was reduced in CAD patients, in association with the lowered mitochondrial respiratory capacity in the EAT and severity of coronary artery stenosis. Our data support the hypothesis that impaired EAT mitochondrial respiratory capacity plays a crucial role in the progression of coronary atherosclerosis, at least in part via a reduced synthesis of adiponectin in the EAT.

## Methods

### Study patients

EAT samples were obtained from 25 patients who underwent elective cardiac surgery with a median sternotomy and cardiac arrest at either of Hokkaido University Hospital or Teine Keijinkai Hospital during the period from July 2015 to August 2016. Patients who had undergone a prior cardiac surgery and patients with chronic inflammatory disease, cancer, or chronic kidney disease with current hemodialysis were excluded.

Of the 25 patients, 12 patients underwent coronary artery bypass surgery and the other 13 patients underwent cardiac surgery for reasons unrelated to atherosclerosis (e.g., mitral valve regurgitation). The 12 coronary artery bypass patients and two of the patients who underwent cardiac surgery other than coronary artery bypass surgery with a history of myocardial infarction were allocated to the CAD group (n = 14). The other patients who underwent cardiac surgery with no coronary artery stenosis (≥75% in at least one vessel was defined as significant stenosis) and with no history of myocardial infarction or percutaneous coronary intervention were allocated to the non-CAD group (n = 11). During the surgery performed for each patient, before the cardiopulmonary bypass was performed, the EAT was excised from the fat depot on the anterior wall near the aortic root within the pericardium.

The study protocol was approved by the ethical committees from the institutions involved (Hokkaido University Hospital and Teine Keijinkai Hospital) and performed according to the Declaration of Helsinki. Written informed consent was obtained from each patient before the surgery. This study was registered in the UMIN Clinical Trials Registry: UMIN000018137.

### Preparation of EAT samples

After EAT was obtained during the surgery, the tissue sample was quickly cut into three pieces. The first piece was transferred on the day of the surgery to an ice-cold relaxing solution (BIOPS, in mmol/L: CaK_2_EGTA 2.77, EGTA 7.23, taurine 20, MgCl_2_ 6.56, ATP 5.77, phosphocreatine 15, dithiothreitol 0.5, 4-morpholineethanesulfonic acid 50, pH 7.1) for the measurement of the mitochondrial respiratory capacity and the mitochondrial reactive oxygen species (ROS) emission in the permeabilized EAT. The second piece of tissue was stored in PAXgene solution (Qiagen, Hilden, Germany) for the later histological analysis. The third piece was frozen in liquid nitrogen and stored at −80 °C for the analysis of the protein content of the adiponectin.

### Mitochondrial respiratory capacity in the EAT

We measured the mitochondrial respiratory capacity of the permeabilized EAT at 37 °C using a high-resolution respirometry (Oxygraph-2k, Oroboros Instruments, Innsbruck, Austria) as described^15,22^. After a careful manual dissection of the capillaries and connective tissues with the use of a magnifying glass, sample tissues (approx. 50 mg) were put into the respirometer chamber filled with 2 mL of MiR05 (in mmol/L: sucrose 110, K-lactobionate 60, EGTA 0.5, 0.1% BSA, MgCl_2_ 3, taurine 20, KH_2_PO_4_ 10, HEPES 20, pH 7.1). Digitonin (2 μmol/L) was added to the chamber to permeabilize the tissue samples.

After the stabilization of baseline respiratory rates, the following respiratory substrates, ADP, and an uncoupler were added in the following order as described^15^: (1) glutamate (final concentration, 10 mmol/l) and malate (2 mmol/L) (complex I-linked substrates); (2) ADP (5 mmol/L); (3) octanoyl-l-carnitine (0.15 mmol/L) (a fatty acid); (4) succinate (10 mmol/L) (a complex II-linked substrate); (5) cytochrome *c* (10 μmol/L); and (6) titration of carbonylcyanide p-trifluoromethoxyphenylhydrazone (FCCP; 0.5 µmol/L increments) (an uncoupler). The integrity of the outer mitochondrial membrane was tested by the addition of cytochrome *c*. An increase in oxygen consumption rate indicates damaged outer mitochondrial membrane because cytochrome *c* does not pass the intact outer mitochondrial membrane^23^, but in this study, there was no increase in oxygen consumption rate after addition of cytochrome *c* in all patients. The respiratory rate (i.e., the O_2_ consumption rate) values are expressed as the O_2_ flux normalized to the permeabilized tissue mass (pmol/s/mg wet weight of EAT).

### Immunohistochemical staining of adiponectin in the EAT

The EAT samples had been fixed with the PAX gene tissue system (Qiagen) and embedded in paraffin. All paraffin blocks were prepared as 5-µm-thick tissue sections. The sections were deparaffinized and heated in citric acid buffer (pH 6.0, 95 °C) for antigen retrieval. After a 10-min incubation with 0.3% hydrogen peroxide, the sections were incubated with anti-adiponectin mouse monoclonal antibody (19F1, ×800, ab22554, Abcam, Cambridge, UK) for 60 min followed by the secondary antibody EnVision (Agilent Technologies, Santa Clara, CA) for 30 min. Next, a brown-colored staining pattern was obtained with the use of 3,3′-diaminobenzidine (DAB) staining. Hematoxylin staining was then added for counter-staining. All incubations were performed at room temperature.

### Protein content of the adiponectin in the EAT

Total protein from the EAT was extracted by using a total protein extraction kit for adipose tissues according to the manufacturer’s instructions (101Bio, Palo Alto, CA). The protein concentration of the adiponectin in the EAT was measured with a Quantikine ELISA kit (R&D Systems, Boston, MA). Simultaneously, the total protein concentration was determined by a bicinchoninic acid assay (Sigma-Aldrich, St. Louis, MO), and the protein concentration of the adiponectin was adjusted for the total protein concentration in the individual tissue samples, expressed as ng/mg total protein. The data of protein content of adiponectin in one CAD patient were not available due to the lack of an EAT sample.

### EAT volume and visceral abdominal fat area

Computed tomography (CT) was conducted in each patient within 1 month before the patient’s cardiac surgery. We estimated the the EAT volume by adding up the EAT area at each axial non-contrast slice multiplied by the slice thickness, using a dedicated offline workstation (Vox-Base; J-MAC System, Sapporo, Japan) as described^24^. The axial continuous slices were chosen for their consistency from the level of the right pulmonary artery splitting from the main pulmonary trunk to the level of the coronary sinus.

In addition, we measured the visceral abdominal fat area with an axial non-contrast CT slice at the patient’s umbilical level using the Vox-Base.

### Gensini score

For the evaluation of the severity of coronary artery stenosis, we used the Gensini scoring system as described^25^. The Gensini score was calculated based on the coronary angiography findings obtained within 1 month before the patient’s cardiac surgery. This score is the sum of all coronary artery segment scores where each segment score equals a predetermined segment weighting factor multiplied by the severity score. Specifically, percentage luminar diameter reductions of 25%, 50%, 75%, 90%, 99%, and complete occlusion were given the severity scores of 1, 2, 4, 8, 16 and 32, respectively. A high Gensini score indicates severe coronary artery stenosis.

### Statistical analysis

Data are expressed as the mean ± standard deviation (SD). We used unpaired t-tests to compare the values of the CAD and non-CAD groups. We examined correlations by performing a linear regression analysis using Pearson’s correlation coefficient. Statistical analyses were performed using GraphPad Prism 7.0a software (GraphPad Software, San Diego, CA), and significance was defined as *P* < 0.05.
